# Effects of Global Postural Re-Education on Pain, Functionality, and Range of Motion in Chronic Non-Specific Neck Pain: A Systematic Review of Randomized Controlled Trials

**DOI:** 10.3390/healthcare13141689

**Published:** 2025-07-14

**Authors:** Philippine Picher, Adérito Seixas, Isabel Moreira-Silva, Joana Azevedo, Ricardo Cardoso

**Affiliations:** 1 FP-I3ID, FP-BHS, Escola Superior de Saúde Fernando Pessoa, 4200-256 Porto, Portugal; 40942@ufp.edu.pt (P.P.); aderito@ufp.edu.pt (A.S.); isabelmsilva@ufp.edu.pt (I.M.-S.); jsazevedo@ufp.edu.pt (J.A.); 2 LABIOMEP, INEGI-LAETA, Faculdade de Desporto, Universidade do Porto, 4200-450 Porto, Portugal; 3 CIAFEL, Faculdade de Desporto, Universidade do Porto, 4200-450 Porto, Portugal

**Keywords:** chronic pain, GPR, joint function, joint mobility, neck pain, pain intensity, posture

## Abstract

**Objective:** Although Global Postural Re-education (GPR) is widely used for musculoskeletal conditions, its specific benefits for this population remain unclear due to inconsistent findings across studies. This systematic review aims to analyze the effects of GPR on pain intensity, functionality, and range of motion (ROM) in individuals with chronic non-specific neck pain. **Methods:** Computerized search was performed in the Cochrane CENTRAL, Lilacs, EBSCO, PEDro, Pubmed, RCAAP and Scielo databases using the keyword combination (“Global Postural Rehabilitation” OR “Global Postural Reeducation” OR “Global Posture Reeducation” OR “Global Postural Re-education” OR “GPR”) AND (“Neck Pain” OR “Cervicalgia”). Methodological quality was assessed using the Physiotherapy Evidence Database Scale. **Results:** Six studies with a total of 393 participants (322 women, aged 18–80) were included. The methodological quality was moderate (average PEDro score: 6.7/10), with frequent limitations related to lack of blinding and allocation concealment. Risk of bias was rated as “some concerns” in four studies and “high” in two. GPR was associated with improvements in pain intensity, functionality, and cervical ROM (flexion/extension). While three studies found no significant differences between GPR and static stretching or specific cervical exercises, the remaining three studies reported greater improvements with GPR compared to manual therapy or traditional neck education and exercise therapy. No adverse effects were reported in any of the included trials. **Conclusions:** GPR appears to be a safe and potentially effective intervention for individuals with chronic non-specific neck pain, particularly in improving pain, function, and cervical ROM. Nonetheless, further high-quality randomized controlled trials are needed to confirm its superiority over other physiotherapeutic interventions and to determine the optimal treatment parameters. PROSPERO registration: CRD420251068974.

## 1. Introduction

Neck pain ranks among the most prevalent musculoskeletal disorders worldwide, contributing significantly to disability, work absenteeism, and substantial social and economic burdens. In the United States, it ranks as the second highest cause of workers’ compensation costs, surpassed only by low back pain [1]. Similarly, in Europe, neck and upper limb musculoskeletal disorders are among the most frequently reported complaints leading to absenteeism and functional limitation [2]. Globally, between 22% and 70% of individuals will experience neck pain at some point in their lives, with its incidence steadily increasing, particularly among those in their fifth decade [3]. Women are disproportionately affected compared to men, and despite favorable outcomes in many cases, recurrence and chronicity rates remain alarmingly high. Approximately 30% of individuals continue to experience symptoms or pain recurrence one year after treatment, underscoring the need for effective long-term management strategies [4]. Chronic neck pain is often categorized as either specific (e.g., traumatic, inflammatory, or degenerative causes) or non-specific, with the latter being the most common presentation in clinical practice [4]. Non-specific neck pain is defined as pain located in the cervical region without an identifiable anatomical or pathological cause, such as trauma, infection, tumor, or radiculopathy. It includes acute, subacute, or chronic presentations and is typically diagnosed when structural abnormalities are not evident through clinical examination or imaging [5]. Non-specific chronic neck pain is frequently associated with reduced motor control, impaired coordination between deep and superficial cervical muscles, and diminished range of motion (ROM), all of which contribute to functional limitations and reduced quality of life [6,7]. These impairments highlight the complexity of managing chronic neck pain and emphasize the importance of addressing both structural and functional deficits in treatment approaches.

Evidence suggests that cervical spine posture plays a critical role in the development and perpetuation of neck pain. For instance, forward head posture—often linked to thoracic kyphosis—has been shown to indirectly affect cervical flexion and rotational ROM, leading to compensatory changes in muscle activation patterns [8]. Prolonged computer work in a seated position exacerbates these postural deviations, promoting functional kyphosis and altering scapular positioning, which increases upper trapezius muscle activity and contributes to muscle imbalances [8,9]. These findings underscore the interplay between posture, muscle function, and pain, highlighting the need for interventions that address these interconnected factors.

Among the therapeutic options available, Global Postural Reeducation (GPR), also known as Rééducation Posturale Globale, has emerged as a promising approach for managing chronic neck pain. Developed by Philippe Souchard in 1981, GPR is a comprehensive stretching technique targeting muscle chains and is widely used in clinical practice across various countries [10]. The technique involves prolonged active postures maintained for 15 to 20 min without allowing compensatory movements, thereby promoting the elongation of shortened muscles and the activation of antagonist muscles [11]. By addressing both muscle tightness and postural alignment, GPR aims to reduce pain, improve functionality, and enhance ROM in individuals with chronic musculoskeletal conditions [12]. Unlike conventional stretching or manual therapy, which typically focus on segmental structures or passive techniques, GPR emphasizes active patient participation, neuromuscular retraining, and global postural correction. The method integrates diaphragmatic breathing, proprioceptive control, and isometric contractions to engage entire myofascial chains. This holistic and individualized approach is theorized to produce more enduring improvements in postural balance, muscle coordination, and pain modulation, distinguishing GPR from traditional physical therapy methods [10].

The clinical effects of GPR have been studied in various conditions, including temporomandibular disorders, chronic neck pain, ankylosing spondylitis, and low-back pain. A literature review from Teodori et al., 2011 [13] identified 13 articles on GPR, of which only four were randomized controlled trials (RCTs) that assessed patient-reported outcomes. Among these, only two RCTs specifically investigated the effects of GPR on chronic neck pain, showing results comparable to other physiotherapeutic interventions. However, methodological limitations in these studies, such as small sample sizes and lack of blinding, highlighted the need for more rigorous research. Subsequent systematic reviews and meta-analyses [12,14] found no significant differences between GPR and other treatments, such as segmental stretching, for musculoskeletal disorders, including chronic neck pain. These reviews also noted the lack of high-quality evidence and the inability to determine whether GPR is better than placebo. More recently, the literature review of Alagingi et al. (2022) [15] compared various postural rehabilitation techniques, including GPR, Pilates, and the McKenzie method, for chronic neck pain. While GPR showed promising results in reducing pain intensity, disability, and kinesiophobia, only one RCT on GPR for nonspecific chronic neck pain was included, underscoring the need for further research. However, existing trials differ substantially in intervention protocols, patient characteristics, and outcome measures, making it difficult to compare results across studies and highlighting the need for a systematic synthesis of the evidence.

Despite its widespread use, the evidence supporting the efficacy of GPR in chronic neck pain remains mixed. This inconsistency in findings underscores the need for a systematic evaluation of the existing literature to clarify the role of GPR in the management of chronic neck pain.

The objective of this systematic review is to analyze the effects of GPR on pain intensity, functionality, and ROM in individuals with chronic non-specific neck pain. By synthesizing the available evidence, this review aims to provide clinicians and researchers with a clearer understanding of the potential benefits and limitations of GPR, ultimately guiding evidence-based decision-making in the treatment of chronic neck pain.

## 2. Methods

### 2.1. Study Design and Search Strategy

This systematic review was conducted in accordance with the Preferred Reporting Items for Systematic Reviews and Meta-Analyses (PRISMA) guidelines to ensure methodological rigor and transparency. The protocol was also registered in the International Prospective Register of Systematic Reviews (PROSPERO) under the registration number CRD420251068974. The PICO framework (Population, Intervention, Comparison, Outcome) was employed to formulate the clinical question:

Population (P): Individuals with non-specific chronic neck pain (pain lasting > 3 months); Intervention (I): Global Postural Reeducation; Comparison (C): Control groups receiving alternative therapies (e.g., static stretching, manual therapy, specific cervical exercises) or placebo; Outcome (O): Pain intensity, functionality, and cervical ROM.

A comprehensive computerized search was conducted across multiple databases, including Cochrane CENTRAL, Lilacs, EBSCO, PubMed, and SciELO, with articles published up to 31 January 2025. The search strategy used a combination of keywords and Boolean operators, such as: (“Global Postural Rehabilitation” OR “Global Postural Reeducation” OR “Global Posture Reeducation” OR “Global Postural Re-education” OR “GPR”) AND (“Neck Pain” OR “Cervicalgia”).

In PEDro database, several searches were performed using the following keywords: “Global Postural Rehabilitation”, “Global Postural Reeducation”, “Global Posture Reeducation”, “GPR”, “Neck Pain” with the option to match all search terms (AND).

In the RCAAP database, the search was conducted using a simplified keyword combination: “Global Postural Reeducation” AND “Neck Pain”, due to platform limitations in handling more complex Boolean expressions or multiple synonym groupings.

Studies were included based on the following criteria: (a) randomized controlled trials (RCTs) comparing GPR to other interventions or control groups; (b) studies enrolling participants with chronic (>3 months) non-specific neck pain; (c) studies published up to 31 January 2025, without restrictions on the starting publication date; (d) studies written in Spanish, French, English or Portuguese; and (e) outcome measures assessing pain intensity, cervical ROM, and/or functionality.

Studies were excluded if they met any of the following criteria: (a) combined GPR with pharmacological treatments or invasive therapies (e.g., injections, surgery); (b) included individuals with acute neck pain (<3 months) or comorbid musculoskeletal conditions (e.g., radiculopathy, temporomandibular joint dysfunction); (c) lacked sufficient details about the intervention (e.g., descriptions of postures, number of sessions, or treatment duration were missing); or (d) had a methodological quality score on the PEDro scale below 5.

### 2.2. Data Extraction

Two independent reviewers (R.C. and P.P.) screened the titles, abstracts, and full texts of the identified studies for eligibility. Discrepancies were solved through discussion or consultation with a third reviewer (A.S.). Data extraction was performed using a standardized form, capturing details on study design, participant characteristics, intervention protocols, comparison groups, outcome measures, and results.

### 2.3. Methodological Quality

The methodological quality of each RCT included in this review was assessed by two independent reviewers (R.C. and P.P.) using the Physiotherapy Evidence Database (PEDro) scoring scale [16]. The PEDro scale consists of a checklist with 11 criteria, of which only 10 are scored. For each criterion met by a study, 1 point is given. Any disagreements in scoring were solved through oral discussions between the reviewers. A consensus was reached on all studies during the first meeting. Studies were classified as having moderate quality if they scored between 5 and 7, and as having high quality if they scored between 8 and 10. According to the PEDro database website, a score of 8 is considered optimal for complex interventions.

### 2.4. Risk of Bias Assessment

The methodological quality of the included RCTs was assessed using the Cochrane Risk of Bias 2 (RoB 2) tool, a revised and validated instrument for evaluating risk of bias in randomized trials [17,18]. This tool evaluates potential sources of bias across five domains: the randomization process; deviations from intended interventions, missing outcome data; measurement of the outcome; and selection of the reported result.

Two reviewers (R.C. and P.P.) independently assessed each study, and disagreements were resolved through consensus. Judgments were categorized as “Low risk”, “Some concerns”, or “High risk” of bias, as per RoB 2 guidelines.

## 3. Results

### 3.1. Study Selection

**Figure 1:**
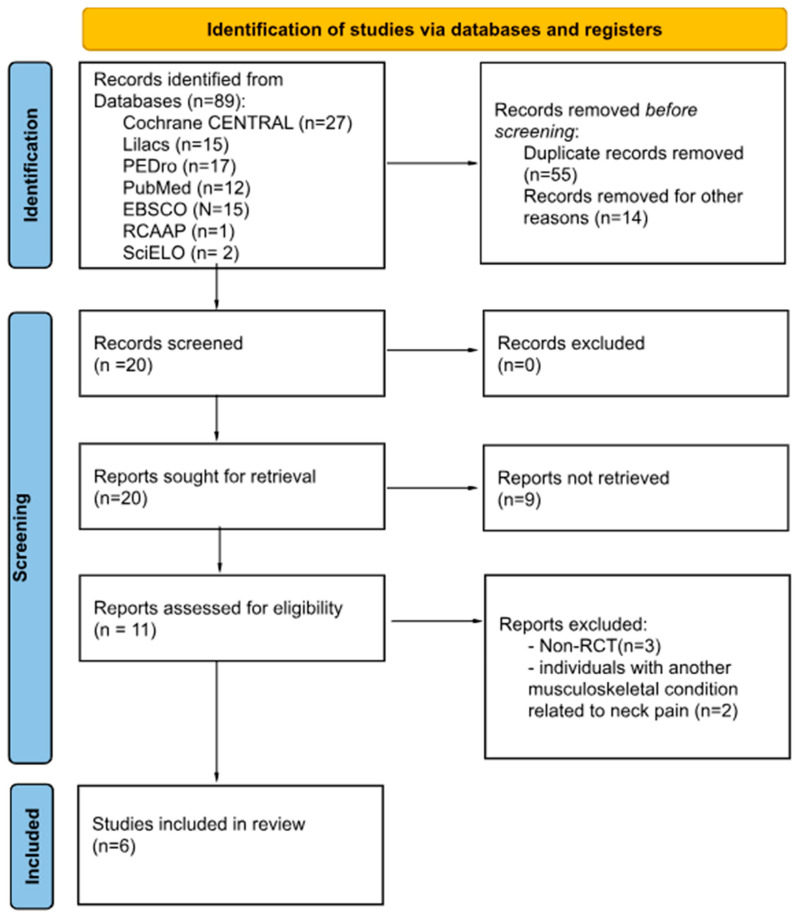
PRISMA flowchart.

The initial database search yielded 89 records, with 55 duplicates and 14 irrelevant studies removed during preliminary screening. After reviewing titles and abstracts of the remaining 20 articles, nine were excluded for not meeting inclusion criteria (e.g., non-RCTs, unrelated topics). Full-text assessment of 11 articles led to the exclusion of five additional studies: three for not being randomized controlled trials [19,20,21] and two for including individuals with musculoskeletal conditions other than chronic non-specific neck pain [11,22]. A total of six studies were included in the final review. The PRISMA flowchart (Figure 1) illustrates the study selection process.

### 3.2. Methodological Quality

The methodological quality of the included studies was assessed using the PEDro scale. The studies scored an average of 6.67 out of 10, indicating moderate quality. None of the studies achieved blinding of participants, therapists, or assessors. All studies met criteria for random allocation, baseline comparability, and intention-to-treat analysis. However, only one study [23] met the criterion for concealed allocation, and one [24] did not meet the criterion for baseline similarity between groups. These findings indicate that, while the included RCTs met several methodological criteria, their overall quality is limited, primarily by the consistent lack of blinding and occasional issues with allocation concealment and baseline equivalence. These limitations should be considered when interpreting the strength of the evidence. The methodological quality scores for each study are summarized in Table 1.

### 3.3. Risk of Bias Assessment

**Figure 2:**
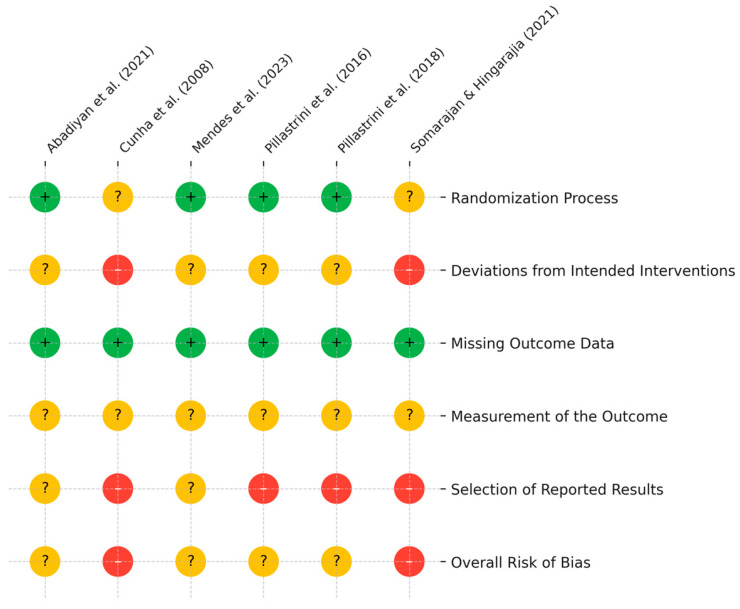
Risk of bias assessment based on the Cochrane Risk of Bias 2 tool [23,24,25,26,27,28]. Symbols indicate the level of bias: low (+), high (–), and some concerns (?).

The risk of bias across the six included studies was variable. A visual summary of the risk of bias across all included studies is presented in Figure 2. While all studies were RCTs and used appropriate designs to compare interventions involving GPR, several exhibited methodological concerns that impact the robustness of their findings.

#### 3.3.1. Bias Arising from the Randomization Process

Most studies reported random allocation to intervention groups. For example, Mendes-Fernandes et al. (2023) [24] and Pillastrini et al. (2016, 2018) [23,27] clearly described the use of randomization software or concealed allocation methods. However, studies such as Cunha et al. (2008) [26] and Somarajan & Hingarajia (2021) [28] did not describe the random sequence generation method or concealment procedures in sufficient detail, raising some concerns of bias. Baseline imbalances were not evident in most studies, and where reported, groups appeared comparable.

#### 3.3.2. Bias Due to Deviations from Intended Interventions

Blinding of participants and personnel was not possible or not reported in most studies, which is common in physical therapy trials. Nevertheless, the risk of bias from deviations is increased when no clear measures are taken to standardize the delivery of interventions or monitor adherence. For instance, Cunha et al. [26] and Somarajan & Hingarajia [28] did not describe whether therapists received training or whether participant adherence was assessed, introducing uncertainty around protocol fidelity. Additionally, intention-to-treat (ITT) analysis was not performed or reported in several trials, which may further compromise internal validity.

#### 3.3.3. Bias Due to Missing Outcome Data

Attrition was low across most studies, with clear reporting of participant flow and reasons for withdrawal when applicable. All studies had complete outcome data or accounted for losses, and no significant differential attrition was detected. This domain was rated as low risk in the majority of included studies.

#### 3.3.4. Bias in Measurement of the Outcome

Outcome measures used across studies were validated tools such as the Visual Analogue Scale (VAS), Neck Disability Index (NDI), and cervical range of motion (ROM) assessed by goniometry or inclinometers. These outcomes are considered objective or minimally susceptible to assessor influence. However, in most studies, outcome assessors were not blinded, which could introduce bias, particularly for subjective outcomes like pain or disability. Given the nature of the interventions and lack of reporting on blinding procedures, several studies received a rating of “some concerns” for this domain.

#### 3.3.5. Bias in Selection of the Reported Result

Selective reporting was a consistent issue. Only one study—Abadiyan et al. (2021) [25]—was registered in a clinical trial database, and registration was completed retrospectively. The remaining studies, including Mendes-Fernandes et al. [24], Cunha et al. [26], and both trials by Pillastrini et al. [23,27], did not mention protocol registration or prespecification of outcomes. Furthermore, none of the studies clearly defined primary versus secondary outcomes in their methods, and no statistical analysis plans were made publicly available. Therefore, the potential for selective reporting of favorable outcomes exists, and this domain was judged as “high risk” in multiple studies.

#### 3.3.6. Overall Risk of Bias

Among the six included studies, four trials—Mendes-Fernandes et al. (2023) [24], Pillastrini et al. (2016) [23], Pillastrini et al. (2018) [27], and Abadiyan et al. (2021) [25]—were judged as having “some concerns” overall. In contrast, Cunha et al. (2008) [26] and Somarajan & Hingarajia (2021) [28] were rated as having a high overall risk of bias, primarily due to limitations in randomization procedures, absence of blinding, and lack of protocol registration. Among the studies with “some concerns,” Mendes-Fernandes et al. (2023) [24] and Pillastrini et al. (2016) [23] stood out as presenting comparatively lower risk, supported by clearer reporting on randomization and outcome completeness. These variations in methodological rigor should be carefully considered when interpreting the overall findings of this review.

### 3.4. Participant’s Characteristics

The six studies included a total of 393 participants, with 322 women and 71 men, aged between 18 and 80 years. Three studies included female individuals, while the others included individuals of both sexes. Of these 393 participants, 365 completed the entire protocol. Of the 28 individuals who did not complete the studies, four dropped out for professional reasons, six for health reasons unrelated to the protocol, six for personal reasons, and 12 for unknown reasons. The number of participants ranged between 33 individuals [26] and 93 [23,27], with an average of 65.5 participants.

### 3.5. Interventions

The studies compared GPR with various interventions, including static stretching [26,28], manual therapy [23,27], a smartphone application for postural correction [25], and specific cervical exercises [24]. GPR interventions typically involved two main postures: (1) the “frog on the floor with arms closed” posture, targeting the anterior muscle chain, and (2) the “frog in the air with arms closed” posture, targeting the posterior muscle chain. Participants maintained each posture for 15 to 20 min, with some studies incorporating a standing posture for postural integration into daily life. The duration of GPR interventions ranged from 4 to 9 weeks.

Follow-up periods varied across studies: no follow-up [24,25,27,28], six weeks [26], and up to six months [23].

### 3.6. Outcomes

#### 3.6.1. Pain Intensity

All studies assessed pain intensity using either the Visual Analog Scale (VAS) or the Numeric Pain Rating Scale (NPRS). The results demonstrated consistent and significant reductions in pain intensity following GPR interventions, with these improvements maintained throughout follow-up periods ranging from six weeks to six months. When compared to alternative interventions, GPR showed comparable efficacy to static stretching and specific cervical exercises without demonstrating superior pain relief [24,26,28]. However, two studies by Pillastrini et al. [23,27] revealed significantly greater pain reduction with GPR compared to manual therapy, with these benefits persisting at the six-month follow-up assessment. Abadiyan et al. (2021) [25] further investigated the adjunctive use of a smartphone application with GPR, finding that this combination produced significantly greater pain reduction than either GPR alone or traditional neck education and exercise therapy administered to the control group. Notably, GPR as a standalone intervention still achieved statistically significant pain reduction relative to the control condition.

#### 3.6.2. Functionality

Five studies assessed cervical functionality using the Neck Disability Index (NDI). The pooled results demonstrated significant improvements in functionality following GPR interventions, with benefits maintained at follow-up assessments. Comparative analysis revealed that, while GPR showed comparable effectiveness to static stretching and specific cervical exercises [24,28], it exhibited superior outcomes relative to manual therapy in one study [27]. Interestingly, Pillastrini et al. (2016) [23] reported that, while GPR showed no immediate post-treatment advantage over manual therapy in reducing disability (NDI), it demonstrated significantly better functional outcomes at the six-month follow-up. The adjunctive use of a smartphone application with GPR [25] produced significantly greater functional improvement than either GPR alone or conventional neck education/exercise therapy. Importantly, standalone GPR still achieved statistically significant functionality gains compared to control conditions across all relevant studies.

#### 3.6.3. Range of Motion (ROM)

Four studies assessed cervical ROM using goniometry. GPR significantly improved ROM in flexion, extension, lateral flexion, and rotation, with sustained benefits observed at follow-up in studies by Cunha et al. (2008) [26] and Pillastrini et al. (2016) [23]. However, GPR was not superior to static stretching or specific cervical exercises in enhancing ROM [24,26]. When compared to manual therapy, GPR demonstrated greater improvements in flexion, extension, and lateral flexion, but not rotation [23]. Pillastrini et al. (2018) [27] further supported these findings, reporting significantly greater gains in flexion and extension ROM with GPR, though no differences were noted for lateral flexion or rotation. Notably, Pillastrini et al. (2016) [23] found that GPR’s benefits in flexion/extension and lateral flexion persisted at six-month follow-up, while rotation improvements remained comparable between groups. The authors suggest these results highlight GPR’s specific efficacy in planes influenced by postural muscle chains.

### 3.7. Adverse Effects

None of the included studies reported any adverse events associated with the GPR interventions. Two trials [23,24] explicitly stated that participants were monitored for adverse effects throughout the intervention period, confirming no events occurred. Two additional studies reported “no adverse events” but did not provide details on their monitoring procedures [25,27]. The last two studies did not explicitly address adverse effects [26,28].

### 3.8. Physiotherapists Experience and Protocol Fidelity

The qualifications and experience of physiotherapists delivering GPR varied across the included studies, potentially influencing treatment consistency and outcomes. One trial explicitly reported that therapists had at least seven years of clinical experience in GPR [24], while others described therapists as “experienced” without specifying the duration of practice [23,25,27]. In Pillastrini et al. (2016) [23], therapists received pre-study calibration sessions to standardize procedures, including agreement on outcome measurement techniques, and ongoing supervision was conducted monthly to ensure fidelity to the GPR protocol.

In contrast, other studies did not provide details regarding therapist training or protocol adherence monitoring [26,28], limiting the ability to assess reproducibility. Notably, Abadiyan et al. (2021) [25] mentioned that participants were guided through each posture by trained physiotherapists who advanced them to their individual limits, suggesting some level of fidelity control during supervised sessions. However, no study reported formal measures of session-to-session technique consistency or participant adherence to prescribed postures outside of supervised sessions.

Overall, while some trials attempted to standardize delivery (e.g., through therapist calibration or supervision), most lacked comprehensive reporting on treatment fidelity, adherence, or fidelity checks, which are essential for evaluating the reliability and replicability of GPR interventions—particularly given its reliance on precise posture execution and prolonged positioning.

Table 2 summarizes the scientific articles included in this review, including the characteristics of the individuals involved in the protocols, the intervention procedures, the parameters and instruments of evaluation.

## 4. Discussion

The objective of this systematic review is to analyze the effects of GPR on pain intensity, functionality, and ROM in individuals with chronic non-specific neck pain. The findings of this systematic review support GPR as an effective therapeutic approach for individuals with chronic non-specific neck pain. Across six randomized controlled trials, most studies reported improvements in pain intensity, functionality, and cervical ROM (flexion and extension) with GPR. Notably, these benefits were sustained over the long term, with follow-ups extending up to six months. This indicates that GPR may lead to lasting changes in postural alignment and muscle balance. While one crossover study reported slightly better outcomes for GPR compared to manual therapy at the six-month follow-up [23,27], these findings should be interpreted with caution as they may reflect differences in exposure time, therapist expectations, or study design rather than intrinsic superiority of the method. In general, the effects of GPR were comparable to those of static stretching and specific cervical exercises, reinforcing the possibility that multiple physiotherapeutic approaches may be similarly effective for this condition [24,26].

The results of this review are in line with previous studies on GPR and other stretching-based interventions for chronic neck pain. The literature review by Teodori et al. (2011) [13] identified only two RCTs that specifically investigated the effects of GPR on chronic neck pain, showing results comparable to other physiotherapeutic interventions. However, the methodological limitations of these studies, such as small sample sizes and lack of blinding, underscored the need for more rigorous research. Subsequent systematic reviews and meta-analyses [11,13] found no significant differences between GPR and other treatments, such as segmental stretching, for musculoskeletal disorders, including chronic neck pain. These reviews also highlighted the lack of high-quality evidence and the inability to determine whether GPR is superior to placebo. More recently, the literature review of Alagingi et al. (2022) [15] compared various postural rehabilitation techniques, including GPR, Pilates, and the McKenzie method, for chronic neck pain. While GPR demonstrated positive effects on pain intensity, disability, and kinesiophobia, only one RCT on GPR for non-specific chronic neck pain was included, further emphasizing the need for additional high-quality studies.

Compared to these earlier reviews, the present study provides several novel contributions. First, it focuses exclusively on chronic non-specific neck pain, while previous reviews included heterogeneous populations or grouped GPR with other interventions without separating diagnoses. Second, this review incorporates recent RCTs—such as those by Mendes-Fernandes et al. (2023) [24] and Abadiyan et al. (2021) [25]—that were not available at the time of prior reviews. Third, a comprehensive methodological appraisal was performed using both the PEDro scale and the Cochrane RoB 2 tool, allowing for a more detailed understanding of study quality and bias. These aspects collectively enhance the clinical relevance, methodological rigor, and contemporaneity of this synthesis compared to existing literature.

The methodological quality, assessed by the PEDro scale, was generally moderate to high. Most studies reported randomization and baseline comparability, but blinding of therapists and assessors was often lacking. Despite these limitations, the overall scores indicate that the evidence is based on acceptable methodological standards, though future trials should improve reporting and assessor blinding.

The Cochrane RoB 2 tool identified several domain-specific methodological limitations across the six included RCTs. Most studies demonstrated low risk of bias regarding missing outcome data, with complete follow-up and transparent attrition reporting. However, concerns were frequent in other domains. The randomization process was often poorly described or lacked allocation concealment (e.g., Cunha [26]; Somarajan [28]). Risk of bias due to deviations from intended interventions was heightened in studies that lacked adherence monitoring and did not implement or report intention-to-treat analyses. Additionally, outcome assessors were rarely blinded, raising concerns about detection bias—especially for subjective outcomes like pain. A critical limitation was selective reporting: only one study [25] was registered in a trial database, and even that occurred retrospectively. The absence of protocol registration or prespecified outcomes in the remaining studies elevated the risk of bias due to selective reporting. These limitations, while not invalidating the findings, underscore the need for more rigorously designed trials with transparent methodology and prospective registration.

Although most included studies reported statistically significant improvements in pain intensity and functionality following GPR interventions, the clinical relevance of these changes must also be considered. For pain intensity, the minimal clinically important difference (MCID) on the Visual Analogue Scale is generally estimated at around 1.5–2.0 cm on a 10-cm scale. Several studies in this review reported Visual Analogue Scale improvements that exceed this threshold, particularly those by Abadiyan et al. (2021) [25] and Pillastrini et al. (2016) [23], suggesting that the effects of GPR may indeed be clinically meaningful. Similarly, for the Neck Disability Index, an MCID of approximately 5–10 points (on a 100-point scale) is often cited. In studies that used the NDI, post-treatment improvements commonly fell within or above this range, indicating potential clinical benefit. Nonetheless, not all studies reported raw score changes, which limits a definitive comparison with established MCID thresholds. Future trials should consistently report absolute changes and compare them against MCID values to better establish the clinical impact of GPR.

### 4.1. Clinical Implications

The integration of digital technologies, such as smartphone applications, into rehabilitation programs has shown promise in enhancing treatment outcomes for individuals with chronic non-specific neck pain. For example, Abadiyan et al. (2021) [25] reported greater improvements in pain, disability, forward head posture, and endurance when a posture-correcting smartphone app was added to a standard GPR program. However, it is important to recognize that these enhancements may reflect not only the effects of GPR itself but also the independent contributions of real-time postural biofeedback, increased self-monitoring, and improved adherence facilitated by the app.

Digital tools like the one used in this study provide patients with reminders, visual feedback, and structured exercise tracking, which can promote better engagement and consistency in performing prescribed exercises at home. These features are known to positively and independently influence rehabilitation outcomes regardless of the specific physical intervention used. Therefore, future research should aim to isolate the relative contribution of each component—GPR alone, app-based biofeedback, or their combination—to determine whether the observed benefits are additive or synergistic.

In addition to technological integration, treatment frequency appears to influence outcomes. Although limited, current evidence suggests that delivering GPR two or more times per week may enhance short-term improvements in pain and function. Notably, Abadiyan et al. (2021) [25], who provided three weekly sessions, observed faster pain reduction compared to other studies using fewer sessions. This finding may indicate a potential dose–response relationship, although further high-quality trials are needed to confirm optimal treatment intensity.

Importantly, GPR demonstrated an excellent safety profile across all included studies, with no adverse events reported. This supports its use as a well-tolerated and feasible intervention for managing chronic non-specific neck pain, particularly when tailored to individual patient needs and preferences.

These findings suggest that GPR can be effectively integrated into clinical practice, especially when combined with strategies that enhance adherence and self-management. The use of technology-enhanced delivery models may offer additional value, particularly for patients who prefer or benefit from digital support systems. However, clinicians should remain cautious in attributing enhanced outcomes solely to GPR without considering the broader context of multimodal interventions.

Furthermore, the clinical implementation of GPR must also take into account its cost-effectiveness, accessibility, and logistical feasibility. Although session durations are generally comparable to conventional physiotherapy, GPR requires delivery by certified and specifically trained physiotherapists, which may limit its availability in some regions. Barriers such as limited access to accredited training and insufficient reimbursement policies in certain health systems may hinder broader adoption. Patient adherence can also pose a challenge as GPR requires active engagement and consistent effort across multiple sessions. These factors should be considered when planning the integration of GPR into clinical pathways or health service delivery models. Despite these challenges, GPR’s favorable safety profile and potential for sustained benefits suggest that, when appropriately implemented, it may offer a positive cost–benefit ratio, particularly for individuals with chronic or recurrent symptoms.

### 4.2. Methodological Considerations—Adherence and Protocol Fidelity

An important methodological limitation identified in this review is the lack of standardized reporting on participant adherence and treatment fidelity across studies evaluating GPR. Given that GPR relies heavily on precise posture execution, prolonged positioning, and behavioral integration into daily life, variations in either patient compliance or therapist delivery may significantly influence outcomes. Only a minority of studies included any form of adherence monitoring. For instance, Pillastrini et al. (2016) [23] instructed participants to perform home exercises twice weekly but did not quantify actual adherence, limiting the ability to assess the relationship between exercise completion and outcome improvements. In contrast, Abadiyan et al. (2021) [25] utilized a smartphone app to track exercise completion, which likely enhanced adherence through features such as reminders and feedback mechanisms, offering a more structured approach to monitoring participant engagement.

Regarding protocol fidelity, although most studies described the core components of the GPR intervention, few provided specific details on whether deviations from the intended protocol occurred during intervention delivery or how adherence to the protocol was ensured. Only Pillastrini et al. (2016) [23] reported pre-study calibration sessions among therapists to standardize procedures, along with monthly supervision throughout the trial to ensure consistency in intervention delivery. Other trials lacked clear descriptions of therapist training, session-by-session verification of technique delivery, or any formal quality assurance checks, making it difficult to determine the extent to which the intervention was delivered as intended.

The absence of detailed reporting on both participant adherence and treatment fidelity limits the ability to assess the reproducibility of GPR effects and raises concerns about the generalizability of findings across different settings and populations. To enhance the scientific rigor and replicability of future GPR research, investigators should incorporate formal measures of both participant adherence and intervention fidelity. These could include structured exercise logs, video recordings of treatment sessions, or digital tracking tools that provide objective data on posture execution and session duration. Such strategies would improve transparency, support replication efforts, and ultimately strengthen the evidence base for GPR in the management of chronic nonspecific neck pain.

### 4.3. Neurophysiological Explanations for GPR’s Effects

The observed benefits of GPR can be explained through several neurophysiological mechanisms that distinguish it from conventional therapies like static stretching or manual therapy. GPR involves prolonged stretching of interconnected muscle chains, which not only activates the inverse myotatic reflex (unlike brief stretches in conventional therapies [29], but also simultaneously addresses compensatory postural patterns.

Additionally, GPR’s 20 min sustained postures induce viscoelastic stress relaxation at a greater magnitude than typical static stretching (often < 60 s), explaining its pronounced effects on tissue stiffness and ROM [30]. Critically, GPR integrates isometric contractions during stretching—a feature absent in passive manual therapy or isolated exercises—which enhances proprioceptive feedback and promotes neuromuscular recalibration [9]. These mechanisms collectively address both peripheral tissue properties and central nervous system adaptations, offering a dual advantage over conventional therapies that target one domain alone.

Although none of the included randomized controlled trials directly investigated these neurophysiological mechanisms, theoretical models and related literature offer plausible explanations. GPR’s combination of prolonged postural loading, active engagement, and respiratory coordination may influence muscle spindle sensitivity, proprioceptive integration, and descending inhibitory control of pain [31,32]. Recent theoretical models propose that chronic musculoskeletal pain involves disrupted sensorimotor integration and altered cortical body representations [33]. Rehabilitation strategies targeting these mechanisms—such as those emphasizing proprioceptive feedback and postural control—may help restore motor function and reduce maladaptive neuroplasticity. These mechanisms could help explain the functional improvements observed in GPR, even when pain intensity changes are modest.

Finally, while chronic neck pain often involves central sensitization [4], GPR’s unique combination of prolonged afferent input (from muscle chains) and efferent reinforcement (via active postural holding) may more effectively modulate nociceptive processing compared to short-duration interventions. This theoretical framework aligns with clinical findings in which GPR outperformed manual therapy in long-term outcomes but showed comparable short-term effects to specific exercises, suggesting its distinct temporal and systemic action.

Furthermore, the improvements in cervical ROM (particularly in flexion and extension) observed in several studies are consistent with the viscoelastic stress relaxation induced by sustained postures in GPR. Similarly, gains in functionality and postural control may be explained by enhanced proprioceptive input and neuromuscular recalibration, while reductions in pain intensity could be associated with central modulation mechanisms activated during active engagement.

### 4.4. Limitations

A relevant limitation of this review is the fact that, although an extensive search was conducted in scientific databases, grey literature was not searched in depth. This may have introduced a degree of bias towards studies with positive findings as these studies are more likely to be published in scientific journals.

The reviewed studies had several limitations that should be considered when interpreting the results. Notably, the experience and training of physiotherapists delivering GPR interventions varied significantly across studies. While some trials employed clinicians with seven years of experience [24], others did not report therapist qualifications [28]. This heterogeneity may influence intervention fidelity, as GPR’s efficacy relies on precise technique execution, and underscores the need for standardized reporting of provider expertise in future trials. Moreover, clinical and methodological heterogeneity of the limited number of included trials limits the comparison of results between trials and generalizability of the results.

Another important factor limiting the interpretation of this review’s findings is the considerable heterogeneity among included studies. Although all trials applied standardized GPR postures, the specific postures varied between studies—including “Frog in the air with closing arms”, “Frog on the ground with opening arms”, and “Standing against the wall with closing arms”. The way these postures were combined, sequenced, or emphasized was not consistent across protocols. Moreover, there was substantial variability in intervention frequency (ranging from once to three times per week), total duration (from four to ten weeks), and the number of sessions (8–12). The comparator groups also differed significantly, comprising segmental stretching, manual therapy, specific cervical exercises, or no treatment. These methodological inconsistencies reduce the ability to make direct comparisons across studies, hinder the identification of the most effective treatment parameters, and preclude quantitative synthesis via meta-analysis.

Additional limitations include the lack of blinding of participants, therapists, or assessors—a common challenge in trials of physical interventions—which may have introduced bias, particularly for subjective outcomes like pain and functionality. Several studies also had small sample sizes, reducing statistical power and generalizability. With the exception of Pillastrini et al. [23,27], intervention durations were brief (4–9 weeks), potentially insufficient for long-term benefits. The included studies had heterogeneous populations in terms of age and sex, which could bias direct inter-study comparisons. Additionally, some studies combined GPR with other interventions, such as manual therapy or cervical exercises, which may have confounded the results.

Furthermore, the overall risk of bias was rated as high in two studies [26,28], primarily due to insufficient information on randomization methods, lack of assessor blinding, and the absence of trial registration or predefined outcome reporting. These methodological flaws may affect the internal validity of their findings and should be considered when interpreting their results within the broader evidence base.

### 4.5. Suggestions for Future Research

To address the limitations of the current evidence base, future research should consider conducting larger, randomized controlled trials with control groups and other intervention groups (e.g., static stretching) to provide more robust evidence on the efficacy of GPR for chronic neck pain. Longer intervention durations and follow-up periods, extending to six months or longer, would help to evaluate the long-term effects of GPR. Investigating the effects of GPR in specific subpopulations, such as individuals with postural abnormalities with chronic neck pain, could help identify patients who are most likely to benefit from this intervention. Additionally, the involvement of experienced GPR physiotherapists (e.g., ≥5 years of practice) should be prioritized. To strengthen the evidence base, future randomized controlled trials should employ prospective registration, rigorous randomization and blinding procedures, and clearly defined outcome measures.

Moreover, future trials should systematically report adherence data and implement fidelity monitoring tools to ensure that GPR is delivered consistently across participants and therapists. This would improve the reproducibility and clinical applicability of the findings.

## 5. Conclusions

This systematic review suggests that Global Postural Reeducation (GPR) is a potentially effective intervention for reducing pain, improving functionality, and enhancing cervical range of motion (particularly in flexion and extension) in individuals with non-specific chronic neck pain. While GPR demonstrated comparable effects to interventions such as static stretching and specific cervical exercises, it may offer advantages over manual therapy and basic neck education, particularly regarding functional outcomes and pain relief.

However, these findings should be interpreted with caution due to several methodological limitations across the included trials, including small sample sizes, short intervention durations, and inconsistent blinding procedures. Additionally, most studies lacked prospective trial registration or prespecified outcome definitions, raising concerns about selective reporting bias. Although one study which employed three sessions per week reported more rapid pain reduction, the evidence is insufficient to establish an optimal treatment frequency.

Importantly, no adverse events were reported in any study, indicating that GPR appears to be a safe and well-tolerated therapeutic approach.

## Figures and Tables

**Table 1:** Methodological quality of included studies (PEDro scale).

Authors (Years)	Criteria										
	2	3	4	5	6	7	8	9	10	11	Total
Abadiyan et al. (2021) [25]	1	1	1	0	0	0	1	1	1	1	7/10
Cunha et al. (2008) [26]	1	1	1	0	0	0	1	1	1	1	7/10
Mendes-Fernandes et al. (2023) [24]	1	1	0	0	0	0	1	1	1	1	6/10
Pillastrini et al. (2016) [23]	1	1	1	0	0	0	1	1	1	1	7/10
Pillastrini et al. (2018) [27]	1	1	1	0	0	0	0	1	1	1	6/10
Somarajan & Hingarajia (2021) [28]	1	1	1	0	0	0	1	1	1	1	7/10

Legend: Criteria (1) Eligibility criteria were specified; (2) subjects were randomly allocated to groups; (3) allocation was concealed; (4) the groups were similar at baseline regarding the most important prognostic indicators; (5) all subjects were blinded; (6) all therapists who administered therapy were blinded; (7) all assessors who measured at least one key outcome were blinded; (8) measures of at least one key outcome were obtained from more than 85% of the subjects initially allocated to groups; (9) all subjects for whom outcome measures were available received the treatment or control condition as allocated or, where this was not the case, data for at least one key outcome was analyzed by “intention to treat”; (10) the results of between-group statistical comparisons are reported for at least one key outcome; (11) the study provides both point measures and measures of variability for at least one key outcome.

**Table 2:** Summary of included studies.

Authors (Year)	Objective of the Study	Sample Characteristics/Study Design	Treatment Method/Treatment Period and Evaluation	Outcomes Measures	Results
Abadiyan et al. (2021) [25]	Evaluate the effects of adding a smartphone application to GPR in individuals with chronic non-specific neck pain.	n = 58 (60, 2 drop-out) 27 M 33 F AA: 28–48 years MA: 38.5 ± 9.1 years G-GPR + App: n = 19 (20, 1 drop-out) Age: 41.3 ± 8.1 years Weight: 63.5 ± 6.6 kg G-GPR: n = 19 (20, 1 drop-out) Age: 40.3 ± 7.9 years Weight: 62.2 ± 7.6 kg G-Control: n = 20 Age: 37.4 ± 9.8 years Weight: 59.8 ± 6.1 kg	Trial Period: 8 weeks 32 sessions: 4 × 50 min/week. G-GPR + App: GPR + App + postural correction at home via App. GPR: 30 min GPR (15 min for each posture) + 15 min cervical exercises and App (Each 5 min) for postural correction. P1: Frog in the air with closing arms. P2: Frog on the floor with closing arms. Postural correction at home: 2 times/day at home during day-off treatments. G-GPR: 30 min GPR (15 min for each posture) + 15 min cervical exercises and postural integration G-Control: Postural correction in DLA’s (“traditional” neck education and exercise therapy).	NDI VAS	Intragroup: G-GPR + App: NDI ↑ (*p* = 0.01), VAS ↑ (*p* = 0.01). G-GPR: NDI ↑ (*p* = 0.04) VAS = (*p* = 0.06). G-Control: NDI = (*p* = 0.41) VAS = (*p* = 0.45) Intergroups: NDI: G-GPR + App > G-GPR (*p* = 0.037). G-GPR + App > G- Control (*p* = 0.001). G-GPR > G-Control (*p* = 0.031). VAS: G-GPR + App > G-GPR (*p* = 0.041) G-GPR + App > G-Control (*p* = 0.001). G-GPR> G-Control (*p* = 0.021)
Cunha et al. (2008) [26]	Compare the effects of SS and GPR in women with chronic non-specific neck pain.	n = 31 (33, 2 drop-out) 33 F AA: 35–60 years MA: Not Available G-GPR: n = 15 MA = 44.4 ± 7.8 years Weight = 62.5 ± 7.1 kg G-SS: n = 16 MA = 48.7 ± 7.3 years Weight = 65.8 ± 8.3 kg	Trial Period: 6 weeks 12 sessions: 2 × 60 min/week during 6 weeks. Follow-up: 6 weeks. G-GPR: 30 min MT + 30 min GPR MT: pompage 3 × 5 breaths GPR: 15 min for each posture P1 and P2. G-SS: 30 min MT + 30 min SS MT: pompage 3 × 5 breaths SS: Passive stretching of the paravertebral, upper limb and cervical muscles.	CROM VAS	Intragroup: G-GPR: Pre-post: VAS ↑ (*p* = 0.000), CROM ↑ (*p* ≤ 0.001) Pre-follow-up: VAS ↑ (*p* = 0.003), CROM ↑ (*p* ≤ 0.013) G-SS: Pre-post: VAS ↑ (*p* = 0.000), CROM ↑ (*p* ≤ 0.001) Pre-follow-up: VAS ↑ (*p* ≤ 0.001), CROM ↑ (*p* ≤ 0.001) Intergroups: VAS: G-SS = G-GPR CROM: G-SS = G-GPR
Mendes-Fernandes et al. (2023) [24]	Compare the effects of GPR in relation to SCE in women with chronic non-specific neck pain.	n= 50 (0 drop-out) 50 F AA: 30–65 years MA: 50.82 ± 8.77 years G-GPR: n = 25 MA = 47.84 ± 8.86 years IMC = 24.02 ± 2.94 kg/m^2^ G-SCE n = 25 MA = 53.8 ± 7.74 years IMC = 24.77 ± 4.08 kg/m^2^	Trial Period: 4 weeks 8 sessions: 2 × 40 min/week G-GPR: 40 min GPR GPR: 15–20 min for each posture (P1 and P2) + 5 min P4 P4: Postural integration in standing position. G-SCE: 40 min SCE MS of cervical deep flexors in SP, and deep extensors in PP. MS of axio-scapular muscles, especially the middle and lower trapezius. Cervical sensorimotor control exercises with visual feedback.	CROM NDI NPRS	Intragroup: G-GPR: CROM ↑ (*p* < 0.001) NDI ↑, NPRS ↑ (*p* < 0.05) G-SCE: CROM ↑ (*p* < 0.001) NDI ↑, NPRS ↑ (*p* < 0.05) Intergroups: CROM, NDI, VAS: G-SCE = G-GPR
Pillastrini & al. (2016) [23]	Compare the effects of GPR with MT in individuals with non-specific chronic neck pain	n = 87 (93; 6 drop-out) 22 M 72 F AA: 18–80 years MA: 47.5 ± 11.3 years G-GPR: n = 43 (46; 3 drop out) MA: 47.5 ± 7.9 years IMC = 24.9 ± 4.3 kg/m^2^ G-MT: n = 44 (47; 3 drop out) MA: 47.4 ± 13.9 years IMC = 24.3 ± 4 kg/m^2^	Trial Period: 5 to 9 weeks + 6 months follow-up. 9 sessions of 60 min 1 assessment immediately after treatment (T1) 1 assessment 6 months after last treatment (T2) G-GPR: 60 min GPR GPR: 20 min for each posture (P1 and P2) + 10 min P4 G-MT: 60 min: Traction and fascia mobilization (30 min) + Passive Mobilization (15 min) + Massage (15 min). Axial cervical traction + mobilization of the cervical fascia. Passive mobilization: Maitland with PA and AP accessory movements: 1 min/cervical level. Therapeutic massage: In the cervical and shoulder areas.	CROM NDI VAS	Intergroups: Pre-post (T1): CROM (F/E, F-lat) VAS: G-GPR > G-MT (*p* < 0.05) NDI, CROM (Rot): G-GPR = G-MT Pre-follow-up (T2): CROM (F/E, F-lat), NDI, VAS: G-GPR > G-MT (*p* < 0.05) CROM (Rot): G-GPR = G-MT
Pillastrini et al. (2018) [27]	Compare the effectiveness of GPR with MT in individuals with non-specific chronic neck pain.	n = 78 (93; 15 drop-out) 22 M 72 F AA: 18–80 years MA: 47.5 ± 11.3 years G- GPR-to-MT: n = 40 (46; 6 drop out) Age: 47.5 ± 7.9 years IMC = 24.9 ± 4.3 kg/m^2^ G- MT-to-GPR: n = 38 (47; 9 drop out) MA: 47.4 ± 13.9 years IMC = 24.3 ± 4 kg/m^2^	Trial Period: 5 to 9 weeks (9 Sessions) + 6 months wash-out + cross-over + 5 to 9 weeks (9 Sessions) 18 Sessions: 2 × 9 sessions of 60 min. Assessment after the last session (T3) 2 groups: G1: MT then GPR; G2: GPR then MT GPR: 60 min GPR 20 min for each posture (P1 and P2) + 10 min P4. MT: 60 min: Traction and fascia mobilization (30 min) + Passive Mobilization (15 min) + Massage (15 min). Axial cervical traction + mobilization of the cervical fascia. Passive Mobilization: Maitland with PA and AP accessory movements: 1 min/cervical level. Therapeutic massage: In the cervical and shoulder areas.	CROM NDI-I VAS	Intergroups: Pre-post (T3): CROM (F/E): G-GPR > G-MT (*p* = 0.0039) CROM (Rot): G-GPR = G- MT CROM (F-lat): G-GPR = G-MT NDI-I: G-GPR > G-MT (*p* = 0.0358) VAS: G-GPR > G-MT (*p* = 0.0006)
Somarajan & Hingarajia (2021) [28]	Compare the effectiveness of GPR with SS in women with chronic non-specific neck pain	n = 61 (64; 3 drop-out) 64 F AA: 18–35 years MA: 24.04 ± 12.02 years G-GPR: n = 32 (31; 1 drop-out) MA: 21.65 ± 3.38 years IMC: 21.74 ± 3.39 kg/m^2^ G-SS: n = 32 (30; 2 drop-out) MA: 26.43 ± 6.07 years IMC: 25.64 ± 4.35 kg/m^2^	Trial Period: 4 weeks 12 sessions: 3 × 60 min/week G-GPR: 30 min GPR + 30 min CT GPR: 15 min for each posture (P1 and P2) + 10 min P4 CT: Isometric cervical strengthening + 20 min wet heat G-SS: 30 min SS + 30 min CT SS: Passive stretching of the paravertebral zone, the MS’s and cervical zone. 2 × 30 s for each segment. CT: Isometric cervical strengthening + 20 min wet heat	NDI VAS	Intragroup: GPR: NDI ↑ (*p* < 0.001), VAS ↑ (*p* < 0.001) SS: NDI ↑ (*p* < 0.001), VAS ↑ (*p* < 0.001) Intergroups: NDI: G-SS = G-GPR VAS: G-GPR = G-SS

Code: ↑: Significant Outcome Improvement =: Without significant change of the Outcome; AA; Age Amplitude, App: smartphone app, AP: Anteroposterior, CROM: Cervical Range of Motion, CT: Conventional Therapy, DLA’s: Daily life activities, F: Female, F/E: Flexion/Extension, F-Lat: Lateral Flexion, G-: Group, GPR: Global Postural Reeducation, M: Male, MA Mean Age, MS: Muscle Strengthening, MT: Manual Therapy, NDI: Neck Disability Index, NPRS: Numeric Pain Rating Scale, PA: Postero-Anterior, P: Posture, Pre-follow up: Comparison of outcomes values between the baseline and immediately after the intervention, Pre–Post: Comparison of outcomes values between the baseline and 6 months after the last intervention, PP: Prone position, ROM: Range of Motion, Rot: Rotation, SCE: Specific Cervical Exercises, SP: Supine Position, SS: Static Stretching, VAS: Visual Analogical Scale.

## Data Availability

No new data were created or analyzed in this study.
